# Spatio-Temporal Constrained Human Trajectory Generation from the PIR Motion Detector Sensor Network Data: A Geometric Algebra Approach

**DOI:** 10.3390/s16010043

**Published:** 2015-12-30

**Authors:** Zhaoyuan Yu, Linwang Yuan, Wen Luo, Linyao Feng, Guonian Lv

**Affiliations:** 1 Key Laboratory of VGE (Ministry of Education), Nanjing Normal University, No.1 Wenyuan Road, Nanjing 210023, China; yuzhaoyuan@njnu.edu.cn (Z.Y.); yuanlinwang@njnu.edu.cn (L.Y.); fenglinyao@gmail.com (L.F.); gnlu@njnu.edu.cn (G.L.); 2 State Key Laboratory Cultivation Base of Geographical Environment Evolution (Jiangsu Province), No.1 Wenyuan Road, Nanjing 210023, China; 3 Jiangsu Center for Collaborative Innovation in Geographical Information Resource Development and Application, No.1 Wenyuan Road, Nanjing 210023, China

**Keywords:** sensor networks, trajectory recovering, geometric algebra, spatio-temporal constraints, trajectory filtering, MERL motion sensor

## Abstract

Passive infrared (PIR) motion detectors, which can support long-term continuous observation, are widely used for human motion analysis. Extracting all possible trajectories from the PIR sensor networks is important. Because the PIR sensor does not log location and individual information, none of the existing methods can generate all possible human motion trajectories that satisfy various spatio-temporal constraints from the sensor activation log data. In this paper, a geometric algebra (GA)-based approach is developed to generate all possible human trajectories from the PIR sensor network data. Firstly, the representation of the geographical network, sensor activation response sequences and the human motion are represented as algebraic elements using GA. The human motion status of each sensor activation are labeled using the GA-based trajectory tracking. Then, a matrix multiplication approach is developed to dynamically generate the human trajectories according to the sensor activation log and the spatio-temporal constraints. The method is tested with the MERL motion database. Experiments show that our method can flexibly extract the major statistical pattern of the human motion. Compared with direct statistical analysis and tracklet graph method, our method can effectively extract all possible trajectories of the human motion, which makes it more accurate. Our method is also likely to provides a new way to filter other passive sensor log data in sensor networks.

## 1. Introduction

Long-term accurate human motion trajectory analysis is becoming more and more important for indoor navigation [1], smart homes [2], behavior science [3], architectural design for buildings [4] and evacuation [5], *etc.* Continuously obtaining human motion data without violating privacy and at low cost is the main challenge for sensor development and data analysis method construction. 

Active sensors (such as RFID tags, WIFI, Bluetooth sensors on mobile devices [6]) and passive sensors (such as cameras [7], passive infrared (PIR) motion detectors [8]) are used widely for human behavior tracking. Active sensors, which send signals on their own initiative, are more accurate in short-time human identification, classification and motion tracking. Active sensors are known to the users themselves and can be used for individual identification. Therefore, they are not suitable for people who are sensitive to privacy intrusions [9]. On the contrary, passive sensors, which only log people’s information when there are people in their range, are used more widely in long-term observation of large scale human indoor movements. The continuous use of a large number of passive sensors can be potentially economical, scalable, efficient, and privacy sensitive in human tracking [10]. A lot of available commercial systems for activity monitoring at home (e.g., Quiet Care Systems, e-Neighbor) are based on these passive sensors. 

Mitsubishi Electric Research Laboratories (MERL) have deployed a large amount (>200) of cheap, energy-efficient and simple passive infrared (PIR) motion detectors to acquire human motion data continuously for about two years [11]. The sensors are densely placed in large areas and log the human motion data continuously on a large scale [8]. Connolly *et al*. used the MERL dataset to model social behaviors including visiting people, attending meetings and travelling with people with the entropy and graph cuts method [12]. They modelled pairwise statistics over the dataset to extract relationships among the occupancy data. The temporal patterns of the human motion in the MERL dataset were analyzed by T-Pattern algorithm [13,14]. Research trying to recover the social networks from the spatio-temporal patterns of the interactions were also developed [15,16]. Because of the special characteristics of MERL dataset, it has been previously used in the IEEE Information Visualization Challenge, and presents a significant challenge for behavior analysis, search, manipulation and visualization.

Different from other passive motion sensors like cameras, the PIR sensor is a binary passive sensor that cannot classify and locate individual human [11]. The PIR sensor works by sensing light emitted in the far-infrared by warm objects and signal on high-frequency changes. If there is anyone moving into the cognitive range, the sensor will be activated and the logged data will change to 1; otherwise the sensor will output 0. Every sensor works continuously to log the time and the active state data as continuous data stream. Each sensor works independently and the sensor does not distinguish the absolute location and the number of people in the area, *i.e.*, one person and several people in the cognitive range will produce the same active 1 output from the sensor. As has been discussed extensively in the literature, it is not possible to log the complete track of people moving around the space using only motion detectors [17]. Although the sensor is related to the geographical locations, only adjacent relations between different sensors can be revealed from the sensor activation log data [18,19]. Therefore, functional mapping and filtering should be applied to transform the observed response sequences back into the spatio-temporal location relations to make the trajectory complete. In the process of the backward mapping from the adjacent relations to absolute spatial coordinates, various spatio-temporal constraints should be integrated in human motion tracking. Because of the complexity of the data, it is hard to solve such high-dimensional and uncertain problems with classical methods. Due to such reasons, the statistical analysis of the human motion pattern from the PIR motion sensors also has considerable uncertainties [14].

It has already been proved that the PIR sensor network cannot provide enough information to recover the trajectory of an individual. Only the statistical behavior pattern can be extracted from the PIR sensor network log data [8]. Technologies such as tracklet graph models were developed to support the dynamic query and visualization of the possible human motion patterns in the spatio-temporal domain [20]. Other methods, including the Kalman filter [21], hidden Markov chain model [2] and topic models [22], are applied to try to extract human motion patterns in a statistical way. However, since the sensor data logged to the human trajectory mapping are not a unique mapping, *i.e.*, the same sensor logging may be caused by different human motions, the accuracy of these existing methods can still been dubious. For example, human guidance and carefully defined tracklet construction rules are required for accurate trajectory visualization [20]. To overcome these drawbacks, some researches try to use multiple device including cameras [17] to help determine the true trajectories, which makes the problem complicated and costly. Since not all the possible human motion trajectories are completely known as the full set, the accuracy of the statistical models may also be problematic. From this perspective, generations of all the possible human trajectory patterns from the sensor log data are important for the sensor data analysis. However, to our best knowledge, there is no method that can retrieve the complete possible trajectories from the PIR sensor network data.

Besides the direct analysis of the human motion patterns from the sensor log data, there’s another way to analyze the human motions from the generation-refinement paradigm. In the generation-refinement paradigm, all the possible human motion trajectories can be firstly generated and then dynamically refined according to the spatio-temporal constraints and sensor activation logs. Then the people tracking problem can be formulated as how to generate all the possible trajectory with several different spatio-temporal constraints according to the sensor activation log. To summarize, the following advantages can be achieved in the human trajectory analysis under the generation-refinement paradigm: (1) the complete possible human motion patterns can be generated; (2) the human trajectory taking place in the geographic space activates the sensor with unique pattern. Mapping from the trajectory to the sensor activation log is unique; (3) both the spatio-temporal correspondence of the sensor activation and the sensor network topology can be used to reduce the uncertainties of the trajectory analysis [17,23]. With the well-designed trajectory generation algorithm, all the possible human motion trajectories can be better extracted and classified.

The key issue of accurate analysis of the human trajectory from PIR sensor networks is how to reduce the uncertainties of the trajectory reconstruction. Several issues should be carefully studied for the human motion analysis using the PIR sensor network data under the generation-refinement paradigm. First, the human motion trajectory is time-varying (*i.e.*, dynamical). Thus, the complete sets of the possible trajectories should be generated dynamically. However, there are rare methods that can support flexible and dynamical trajectory generation according to the sensor network topologies. Second, both the sensor network topology and the sensor activation log data should be formulated as spatio-temporal constraints, but the sensor activation log data and the spatio-temporal constraints data are significantly different. Few method can support the unified representation of both the sensor activation log data and spatio-temporal constraints. Third, the formulated spatio-temporal constraints should be dynamically integrated into the trajectory generation to refine the trajectories. Yet, hardly is there any method that can support such integration of the complex spatio-temporal constraints with the dynamical trajectory generation.

To overcome the above problems, we developed a new GA-based method to refine the human motion trajectories in this paper. At first, the mathematical definition of the geographical network, sensor activation response sequences and the human motion are defined under the unified GA framework. The relations among the three are analyzed. Then a GA-based dynamical trajectory generation process is defined to generate all the possible human motion trajectories according to the sensor network topology. By integrating both the temporal and spatial constraints, which are extracted from the sensor activation log and predefined rules during the trajectory generation, all the possible human motion patterns that satisfy both the temporal and spatial constraints are dynamically generated. Finally, a complete algorithm to extract all the possible human motion trajectories are proposed. The algorithm is applied to the MERL datasets to evaluate the correctness and performance.

The paper is organized as follows: the problem definition and basic ideas are described in Section 2. The methods, including the human trajectory generation and refinement algorithm, are described in detail in Section 3. The case study and the performance analysis are given in Section 4. Discussion and conclusions are given in Section 5.

## 2. Problems and Basic Ideas

### 2.1. GA and GA Representation of PIR Sensor Networks

In the whole trajectory analysis, there are several concepts that should be defined and analyzed. These are geographical network, sensor activation response sequences, and human behavior semantical sequences. Geometric algebra (GA), founded on the dimensional computation, is an ideal tool for the multidimensional algebraical element representation [24,25,26,27,28,29]. Under the GA framework, any network topology can be mapped into a special Clifford Algebra space Cl(n), and the fundamental elements of the network (*i.e.*, nodes, edges and routes) can be coded as algebraic elements (*i.e.*, blades and multivectors) of this mathematical space [24]. Then the route can be dynamically generated according to the GA products using matrix multiplication [26]. With well-defined computation mechanism, the algebraic network computation can make the route generation and analysis symbolically with low complexities [26,29]. The constraints as well as multi-constrained routing can also be achieved under the GA framework [30,31]. With the GA-based network presentation, the construction of the network expression and calculation model, where there is a unified relationship among the network expression, relation computation and the path search, can be achieved.

Given any positive number *n* > 0, the Clifford algebra/Geometric Algebra system Cl (n) can be generated by the vector set $\{f_{i}\},\;1\leq i\leq n$. The elements of the Clifford algebra space Cl (n) are:

(1) $$\left\{ \begin{array}{l} scalars:f_{0}=1\in R\\ vectors:f_{1},\ldots ,f_{n}\\ bivectors:f_{i}f_{j}=f_{ij},0<i<j<n\\ \vdots \\ n-vectors:f_{1}f_{2}\ldots f_{n}=f_{12\ldots n} \end{array}\right.$$

**Figure 1:**
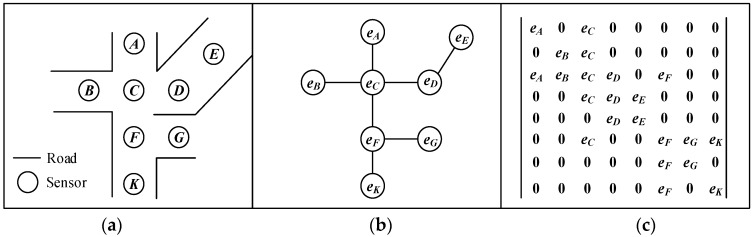
Definition of the geographical sensor network. (**a**) Geographic Secsor Distribution; (**b**) Geographic Secsor Network; (**c**) Adjacent matrix *M*.

Assuming G(V,E) is a graph that have n nodes, we can code each node as an individual algebraical basis of a special Clifford space Cl (n). Given $e_{i},1\leq i\leq n$ be the basis vectors of the Cl (n), the metric matrix which determined by the GA adjacent matrix of the network can be formulated as:

(2) $$A[i,j]=\{\begin{array}{ll} e_{ij} & \mathrm{if}\;(v_{i},v_{j})\in E\\ 0 & \mathrm{otherwise} \end{array}$$

where the element $A[i,j]=e_{ij}$, which represents the edge from the node *i* to the node *j*, is the *i*-th row and the *j*-column of the geometric adjacent matrix. A[i,j]=0 means there is no edge connected from the node *i* to the node *j*. According to the definition, we can formally define the geographical sensor network as follows:

*Definition 1: The Geographical sensor network*. The geographical sensor network is a physical geographical space where the sensors are located. In this sensor network, each sensor represents one node of the network. The sensors are only connected with the adjacent node according to the geographical spatial topology. A typical representation of a geographical sensor network is depicted in Figure 1.

Since the geographical space can be indicated by the network structure of the geographical sensor network (Figure 1a), it is possible to directly represent the geographical space using the GA-based network representation [24,25]. Here, we code each sensor in the geographical sensor network as an individual vector basis (Figure 1b), and then the sensor network can be represented using the GA adjacent matrix ***M***. The adjacent relations between different nodes are inherited in ***M***. To make the computation more efficient, only the network node of the route is logged in the adjacent matrix ***M***. For example, if *e_i_* and *e_j_* are connected, the *i*-th row and *j*-th column element of the adjacent matrix ***M*** is logged as elements of *e_j_* (Figure 1c). Because the sensor distribution is dense, no route weights are required to be stored in the adjacent matrix. As Figure 1c shows, the route in the sensor network can be represented as blades [26]. According to the construction rule, the adjacent network structure is directly correspondent to the adjacent matrix. All the routes between the sensors are logged in the elements with certain grade of the adjacent matrix. Therefore, the trajectory construction can be seen as the multiplication of the adjacent matrix. The spatio-temporal constraints can also be applied to filter the trajectories during the adjacent matrix multiplication. 

The PIR sensor network is a set of PIR sensors installed with the intention to cover the floor area completely with little or no overlap between the sensor viewing fields. Assuming there is a PIR sensor i located in the place with a coordinate of *L*(*X_i_*,*Y_i_*), the output of this sensor in the time period from 1 to *t* is a time series with a binary active state $D=\{x_{1},x_{2},\cdots ,x_{t}\}$, where:

$$x_{i}=\left\{ \begin{array}{l} 1,\;the\;sensor\;is\;active\\ 0,\;otherwise \end{array}\right.$$

Given a PIR sensor network composed of n sensors with each sensor having its own location *L*, which can be expressed as *L* = {*L*_1_,*L*_2_,*L_i_*,…,*L_n_*}. The state of the PIR sensor network at time instant *t* can be seen as an observation, therefore all the observation time can be expressed as *T* = {*t*_1_,*t*_2_,*t_j_*,…,*t_m_*}, and the state series of each time instant can produce a state of the whole sensor network. The observation, notated as *X*, can thus be encoded as a binary set as *X* = {0,1}, where 1 means the corresponding sensor is active, 0 otherwise. For the given sensor located at *L_i_*, the observation can be represented as a feature vector $O(L_{i})=\{X_{t_{1}}^{L_{i}},X_{t_{2}}^{L_{i}},\cdots ,X_{t_{j}}^{L_{i}},\cdots ,X_{t_{m}}^{L_{i}}\}$; Similarly, the feature vector at an instant time $t_{j}$ can be encoded as $O(t_{j})=\{X_{t_{j}}^{L_{1}},X_{t_{j}}^{L_{2}},\cdots ,X_{t_{j}}^{L_{i}},\cdots ,X_{t_{j}}^{L_{n}}\}$. Therefore, the observation of all the sensors during all the time can be expressed as a feature matrix $O(L,T)=\{X_{t_{1}}^{L_{1}},X_{t_{1}}^{L_{2}},\cdots ,X_{t_{j}}^{L_{i}},\cdots ,X_{t_{m}}^{L_{n}}\}=\sum_{j=1}^{m}\sum_{i=1}^{n}X_{t_{j}}^{L_{i}}$. If there is any person moving in the PIR sensor network area, a trajectory as location series $P=\{P_{j}\}=\{P_{t_{1}},P_{t_{2}},\cdots ,P_{t_{j}},\cdots ,P_{t_{m}}\}$ can be logged by the sensor network. With this people motion trajectory, a corresponding observation feature vector series $\left\{X_{p}\}=\{X_{t_{1}}^{L_{P_{1}}},X_{t_{2}}^{L_{P_{2}}},\cdots ,X_{t_{j}}^{L_{P_{j}}},\cdots ,X_{t_{m}}^{L_{P_{m}}}\right\}$ can be outputted from the sensor network. In the feature vector series, there exist $X_{t_{j}}^{L_{P_{j}}}=1$, it means that the person/people are located in the cognitive range of sensor $L_{P_{j}}$ at time $t_{j}$. Since the people are walking in the geographical space time, the observation feature vector series should be constrained by several spatio-temporal constraints {C}. For example, people’s movement should be constrained by the spatial structure (*i.e.*, topology) of the PIR sensor network, *i.e.*, the person/people cannot move between none adjacent sensors. Similarly, there are also temporal constraints that should make the time intervals between different motions acceptable. e.g., the human motion will not exceed the maximum possible velocities.

**Figure 2:**
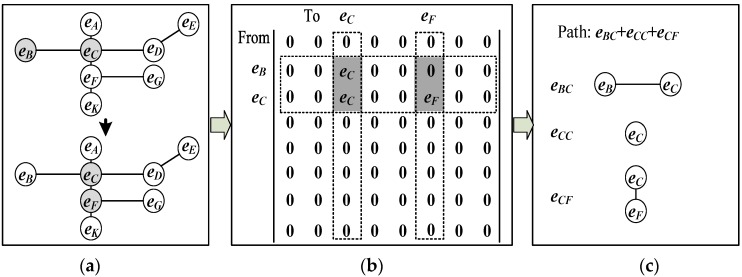
Definition of the activation response network. (**a**) Activated sequences of sensors: $\left\{e_{B},e_{C}\}\to \{e_{C},e_{F}\right\}$; (**b**) Response matrix $M_{F\{e_{B},e_{C}\}T\{e_{C},e_{F}\}}$; (**c**) The possible paths.

Since the PIR sensor only logs the binary response of certain locations and the response time of different responses between different sensors, which can be seemed as activated sequences. It is also possible to represent the sensor activation log as blades. With these blades, the sensor activation sequence can further be projected into the network space. In this way, network structures can be extracted from the sensor activation sequence. So, with the sensors coded with the GA space basis, we define the sensor activation response network as follows:

*Definition 2: The sensor activation response network*. A sensor activation response network is a possible sub-network of the response from the neighbor sensors of activated sensors. For example, in the Figure 2a, if the sensors $\left\{e_{B},e_{C}\}\to \{e_{C},e_{F}\right\}$ is one of the activated sequences within acceptable time constraints, the response network can be defined as the sub-network of the original network from nodes $e_{B},e_{C}$ to $e_{C},e_{F}$. As shown in Figure 2b, the response adjacent matrix $M_{F\{e_{B},e_{C}\}T\{e_{C},e_{F}\}}$ takes the $e_{B},e_{C}$ rows and $e_{C},e_{F}$ columns of the network matrix ***M***, which means the path begins from the sensor *C*,*F* and ends at the sensors *B*,*C*. From the matrix the possible paths can be also estimated as shown in the Figure 2c.

The sensor activation response network can be seen as the representation of the spatial constraints of the PIR analysis. It is not possible to directly move a person from one sensor to a non-adjacent sensor in the geographical space. Therefore, it is impossible to construct a real trajectory to represent the human motion, so in this paper, we think it is not suitable to consider the sensor activation sequence between two non-adjacent sensors as a single trajectory, but a combination of several trajectories.

In a sensor activation response sequence, the two adjacent sensor activities must happen in acceptable time intervals [32]. Because the human motion may end at a certain sensor, the sensor itself cannot distinguish the individuals from each other. The sensor activation with a big time difference may be caused by different people. With these assumptions, the determination of the starting and ending nodes of a trajectory depends on the spatial and temporal intervals to process the sensor activation response sequence. The sensor activation response sequence is caused by the human motion trajectory, which is also connected node-by-node in the network. Therefore, we can define the human motion trajectory similarly to the sensor activation sequence. The human motion sequence is defined as follows:

*Definition 3: The human motion trajectory sequence.* The human motion trajectory sequence is a sequence that represents the human motion structure from any sensor node to another sensor node in the geographic spatio-temporal space. The human motion trajectory sequence is an orderly sequence that can also be represented as a series path matrix, which applied the oriented join product of timely-adjacent response matrix according to the real human motion.

Taking the sensor network in the Figure 1 as an example. Assuming there are activate sensor sequences $\left\{e_{B},e_{C}\}\to \{e_{C},e_{F}\}\to \{e_{A},e_{G}\right\}$, then two response networks can be constructed by $\left\{e_{B},e_{C}\}\to \{e_{C},e_{F}\right\}$ and $\left\{e_{C},e_{F}\}\to \{e_{A},e_{G}\right\}$. To better represent the path extension using Clifford algebra, we extend the oriented join product to the matrix (notated as oriented join matrix product ∪). The ‘∪’ is defined as:

(3) $$\left|\begin{array}{ccc} a_{11} & \ldots & a_{1n}\\ \vdots & \ddots & \vdots \\ a_{m1} & \cdots & a_{mn} \end{array}\right|\cup \left|\begin{array}{ccc} b_{11} & \ldots & b_{1n}\\ \vdots & \ddots & \vdots \\ b_{m1} & \cdots & b_{mn} \end{array}\right|=\left|\begin{array}{ccc} a_{11}\cup b_{11} & \ldots & a_{1n}\cup b_{1n}\\ \vdots & \ddots & \vdots \\ a_{m1}\cup b_{m1} & \cdots & a_{mn}\cup b_{mn} \end{array}\right|$$

Therefore, the path matrix can be calculated by the equation:

(4) $$\begin{array}{cc} A_{\left\{e_{B},e_{C}\to e_{C},e_{F}\to e_{A},e_{G}\right\}} & =M_{F\{e_{B},e_{C}\}T\{e_{C},e_{F}\}}\cup M_{F\{e_{C},e_{F}\}T\{e_{A},e_{G}\}}\\ & =\begin{array}{l} e_{B}\\ e_{C} \end{array}\overset{\begin{array}{cc} e_{C} & e_{F} \end{array}}{\left|\begin{array}{ll} e_{C} & 0\\ e_{C} & e_{F} \end{array}\right|}\cup \begin{array}{l} e_{C}\\ e_{F} \end{array}\overset{\begin{array}{cc} e_{A} & e_{G} \end{array}}{\left|\begin{array}{ll} e_{A} & 0\\ 0 & e_{G} \end{array}\right|}=\begin{array}{l} e_{B}\\ e_{C} \end{array}\overset{\begin{array}{cc} e_{A} & e_{G} \end{array}}{\left|\begin{array}{ll} e_{CA} & 0\\ e_{CA} & e_{FG} \end{array}\right|} \end{array}$$

Then the possible human motion trajectory sequence can be calculated as: $P_{\left\{e_{B},e_{C}\to e_{C},e_{F}\to e_{A},e_{G}\right\}}=e_{BCA}+e_{CCA}+e_{CFG}$. Since each trajectory segment for the real human motion can be seen as a blade and the whole motion trajectory sequences can be seen as linkers between different motion trajectory segments, we can apply the oriented join product to generate all possible connections between trajectory segments [24]. In this way, we can reconstruct and filter the sensor activation response sequences to analyze the human motion trajectory by using algebraic calculation.

### 2.2. The Problems of Trajectory Generation from PIR Sensor Networks

**Figure 3:**
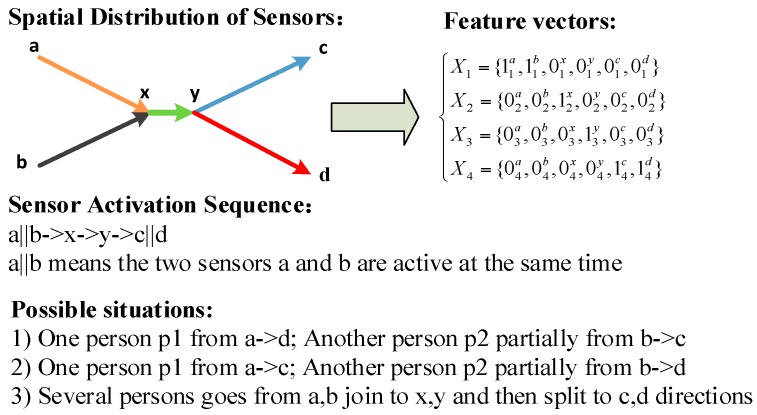
The uncertainty of the sensor response.

According to the characteristics of the PIR sensor, the mapping {L}→{X} is a unique one-to-one mapping, *i.e.*, the same human trajectory or trajectory combinations will definitely produce the same sensor network activation series. However, the binary sensor activation characteristics of the PIR sensor makes the sensor only able to log the human passing states but not the individual information. If there is only one human motion, the sensor activation sequence (*i.e.*, the feature vector) directly corresponds to the trajectory and activation sequence in the time domain. Both the spatial topology of the sensor network and the human trajectory can be revealed from the sensor activation sequence [27]. However, if there are more than one trajectory made by different people, we cannot classify the spatio-temporal correlations and correspondence between different sensor responses according to the sensor log. In this situation, the inverse mapping {X}→{L} may not be unique, *i.e.*, the same sensor activation observation may indicate different human motion trajectories. What’s worse, since different trajectories can be intersected, it is not easy to directly determine the starting and the ending nodes of a certain trajectory. A typical example of this uncertainty is illustrated in the Figure 3. With the same sensor activation sequence, different trajectory patterns are likely to be revealed. Then the human motion trajectory analysis problem under the generation-refinement paradigm can be formulated as how we can extract all the possible trajectories {*L*} from the sensor activation data {*X*} according to both the temporal and spatial constraints *C*.

### 2.3. Basic Ideas

According to the above problem definition, the sensor network can be seen as a geographical network with each sensor being one node of such a network. Because the sensors do not overlap, the sensor activation only affects the adjacent sensor. In addition, the sensor node is also a construction node of the human motion trajectories, *i.e.*, the sensor node in an individual trajectory is adjacent to and only affects the adjacent sensor node. Because each individual human motion has unique map to the activation of the sensor logs, the human motion is restricted by the spatial topology of the real sensor distribution. Therefore, the trajectory construction can be seen as the route generation one node by one node. The sensor activation sequences are not only a fusion of several human trajectory motions, but also the spatial and temporal constraints that will limit the possible and impossible trajectories in the geographical spatio-temporal space. Therefore, the human trajectory analysis can be split into two steps: (1) dynamically generate all possible routes for human trajectories node by node; (2) refine/filter the possible human trajectories to make the trajectory consistent with the sensor activation log data and other spatio-temporal constraints. In this way, the problem can be solved in the generation-refinement paradigm.

According to the generation-refinement paradigm, the problem can be further decomposed into the following sub-problems: (1) how to represent and link both the sensor networks and trajectories in the same unified mathematical framework; (2) how to dynamically generate the trajectories according to both the spatial network topology constraints and the temporal constraints from the sensor log data; (3) how to determine the starting and the ending nodes of each individual human motion trajectory. To deal with the sub-problems (1) and (2), we should develop a flexible mathematical expression framework that can represent both the network and human trajectories. Not only the nodes, routes and whole networks should be represented using the same paradigm of mathematics, but also the representation should support the dynamical route generation node by node in the network. In addition, the spatial and temporal constraints should also be represented with the same mathematical tool and integrated in the dynamical route generation. For sub-problem (3), we should determine certain rules that can be used to classify the trajectories to determine the starting and the ending nodes.

**Figure 4:**
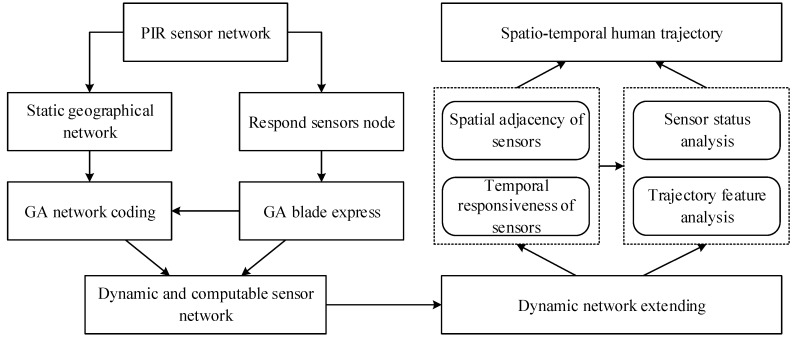
Basic idea.

With the advantages of the spatio-temporal representation properties of GA, we modeled the whole PIR sensor network by GA network coding. The geographical space is first defined and represented as a geographical sensor network. The sensors are coded as the geometric basis of the high dimensional GA space. Then the interaction and response sequence can be embedded as blades. These blades can then represent both the possible human motion trajectories and the sensor activation sequence. By integrating constrains from the real sensor activation observational data, the possible human motion trajectories can be dynamically generated. The overall framework of our basic idea is illustrated in the Figure 4.

## 3. Methods

### 3.1. Classification of Sensor Status According to the Trajectory

**Figure 5:**
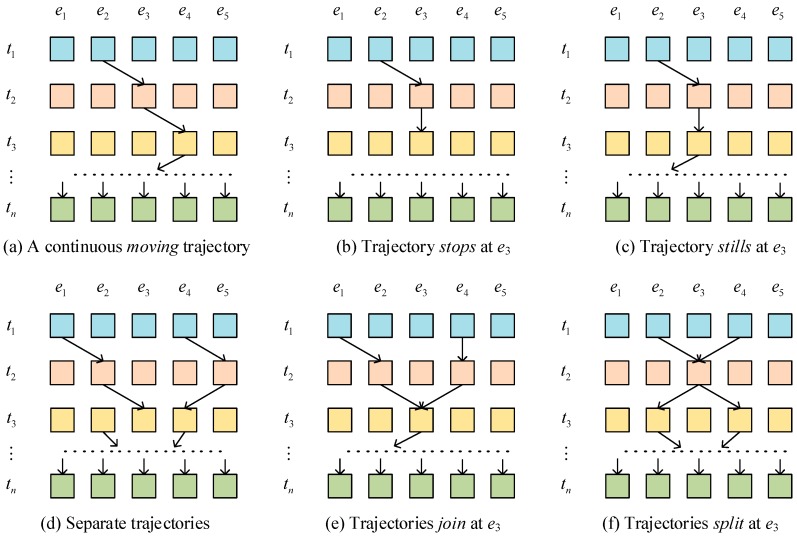
Different situation of the trajectory tracking.

Different from classical network analysis, which has determined the starting and the ending nodes, the ending node during the trajectory tracking is not constant but changed according to the human motion. Since there is more than one trajectory in the geographical space, the classification of different trajectories is important in our analysis. By considering the trajectory generation as a trajectory tracking problem from the dense adjacent matrix, several different situations can be revealed according to the sensor activation log [20] (Figure 5). Both the tracking of the individual trajectory and multiple trajectories are considered. For individual trajectory, the sensors in the trajectory can be classified into three different states: moving, stop and still (Figure 5a–c). The moving status means the trajectory will lead an adjacent sensor to continuously extend the trajectory. The sensor activation sequences will be: the sensor is active in a short time and then the adjacent sensor is activated. The stop states means the trajectory ends at the senor node. In this condition, the sensor is continually active, but after a certain period, there’s no adjacent sensor active. The still status means the person stays in the range of certain sensor for a long time, but this sensor is not stopped. Therefore, we cannot make that sensor a stop node of its trajectories. The data sequence should be: the sensor is continuously active for a time period and then after a period, the adjacent sensor is activated, and the trajectory continues.

To classify the sensor states of the still and stop nodes is important in the trajectory tracking, because it relates to the starting and ending nodes of the trajectories in the foundation of the trajectory generation/tracking. However, it is not an easy task to simply distinguish the two states directly from the sensor activation data. Since the trajectory stills at a node for a very long time, it can be seen that the trajectory stops at the node and a new trajectory is then started from the same sensor node. A more practical way to classify the two states should be defined at a certain time interval of *t* to determine the granularity of the trajectory segment. In this way, if the stay time in one sensor is larger than *t*, we can classify them into the state of stop. And we assume the next trajectory starts from the current sensor. With this configuration, the tracking for individual trajectories can be simply achieved by direct adjacent matrix multiplication [25,26,28,30].

In most common conditions, there should be more than one trajectory in the sensor networks. With more than one trajectory the sensor activation status becomes complex. The simplest condition is that the trajectories are not intersected (Figure 5d). These two non-intersected trajectories can be seen as two separated individual trajectories. However, there will be people from two distinct trajectories joining into one single trajectory (Join, Figure 5e). And there are also people who are originally in a single trajectory but separated into several trajectories (Split, Figure 5f). To the responses of the sensor network, the join condition will lead to several non-adjacent sensor activities, which then will be combined into one common sensor, and only adjacent sensors of the join sensor will be activated afterwards, thus indicating the two different trajectories are combined into a single trajectory. In the split situation, the sensor active trajectory is a single trajectory that only the adjacent sensor is activated, and after a certain sensor, the trajectory splits and two or more non-adjacent sensors are activated, which means the trajectories are split from one.

As the human motion trajectory sequence can be expressed and calculated in the GA space, we can also construct the GA-based classification method of trajectories. Given the sensor activation sequences $\left\{e_{A},e_{B}\}\to \{e_{C},e_{D}\}\to \{e_{E},e_{F}\right\}$, the associated human motion trajectory can be calculated as:

(5) $$\begin{array}{cc} A_{\left\{e_{A},e_{B}\to e_{C},e_{D}\to e_{E},e_{F}\right\}} & =M_{F\{e_{A},e_{B}\}T\{e_{C},e_{D}\}}\cup M_{F\{e_{C},e_{D}\}T\{e_{E},e_{F}\}}\\ & =\begin{array}{l} e_{A}\\ e_{B} \end{array}\overset{\begin{array}{llll} e_{C} & & & e_{D} \end{array}}{\left|\begin{array}{ll} \alpha_{11}e_{C} & \alpha_{12}e_{D}\\ \alpha_{21}e_{C} & \alpha_{22}e_{D} \end{array}\right|}\cup \begin{array}{l} e_{C}\\ e_{D} \end{array}\overset{\begin{array}{llll} e_{E} & & & e_{F} \end{array}}{\left|\begin{array}{ll} \beta_{11}e_{E} & \beta_{12}e_{F}\\ \beta_{21}e_{E} & \beta_{22}e_{F} \end{array}\right|} \end{array}$$

where **α** and **β** can take the value 1 or 0, which indicate the connectivity of sensors, for example: if **α**_11_ = 0, sensors *A* and *B* are not adjacent. Therefore, the trajectories structures are depending on the values of these two adjacent matrix:

(6) $$m_{1}=\left|\begin{array}{ll} \alpha_{11} & \alpha_{12}\\ \alpha_{21} & \alpha_{22} \end{array}\right|,m_{2}=\left|\begin{array}{ll} \beta_{11} & \beta_{12}\\ \beta_{21} & \beta_{22} \end{array}\right|$$

(1) Continuous *moving* trajectory

As the continuous *moving* trajectory is extended with no branch, the path can only have a single starting node and ending node. So, the response matrixes must be the diagonal matrix that avoids having two or more nonzero value in one row or column. The typical expresses of adjacent matrix are:

(7) $$\left\{ \begin{array}{l} m_{1}=\left|\begin{array}{ll} 1 & 0\\ 0 & 1 \end{array}\right|,m_{2}=\left|\begin{array}{ll} 1 & 0\\ 0 & 1 \end{array}\right|\\ m_{1}=\left|\begin{array}{ll} 0 & 1\\ 1 & 0 \end{array}\right|,m_{2}=\left|\begin{array}{ll} 0 & 1\\ 1 & 0 \end{array}\right| \end{array}\right.$$

According to the Equation (4), the trajectories can be calculated as $e_{ACE}+e_{BDF}$ or $e_{ADF}+e_{BCE}$. The result shows that the paths are continuous moving trajectories and separate trajectories.

(2) *Join* and *split* trajectories

Unlike the Continuous moving trajectory, the *join* and *split* trajectories need the path that have the two or more starting nodes or ending nodes. Therefore, the typical expresses of adjacent matrix are:

(8) $$\left\{ \begin{array}{l} m_{1}/m_{2}=\left|\begin{array}{ll} 1 & 1\\ / & / \end{array}\right|\begin{array}{cc} & join\;trajectories \end{array}\\ m_{1}/m_{2}=\left|\begin{array}{ll} 1 & /\\ 1 & / \end{array}\right|\begin{array}{cc} & split\;trajectories \end{array} \end{array}\right.$$

where “/” means the element can be 1 or 0, which will not influence the results. As Equation (8) shown, only if $m_{1}$ or $m_{2}$ has two “to” nodes, the human motion paths will result in *join* trajectories; only if $m_{1}$ or $m_{2}$ has two “from” nodes, the human motion paths will result in *split* trajectories.

(3) *Still* and *stop* nodes

According to the GA-based trajectory representation methods, the trajectory with *still* nodes can be expressed as $e_{x_{1}x_{2}\cdots cc\cdots x_{n}}$, and *e_c_* is the *still* node. The repetitions of *e_c_* suggest the duration time of the *still* node. If the duration time bigger than predefined threshold value, *e_c_* can be also seemed as the *stop* node. Therefore, the most important is to identify the *e_cc_* structure in trajectory sequence. According to the definition of human motion trajectory sequence, if there is a diagonal element (which has the same row and column number) in response matrix, this paths must exist a *still* node.

In a common situation of the human motion, the adjacent sensors will be active and respond continuously. With continuous sensor response log, we can track the spatio-temporal correlations between different sensors in the geographical space and then transform them into possible trajectories. In this paper, we assume that a typical human walks at a velocity of 1.2 m/s. Since no gap exists between different sensors, a human passing through a sensor may need a response time of 2–4 s. So we select the median time window of 3 s to do the object tracking. If any object activates a sensor and activates the next adjacent sensor within the next node, it will probably be a trajectory. If there is no adjacent node activated, then we can consider the node the ending node of a trajectory.

### 3.2. The Generation of All Possible Trajectories

We refer to the generative route construction method to extend the possible path according to the sensor network topology as well as the sensor log data. Since the route generation in the GA space may produce redundant or impossible routes in real geographical space, spatial and temporal constraints to each generation are applied firstly to refine the generated trajectories. Based on the spatial object tracking idea, we propose the following route generation and constraints filtering method.

(1) The trajectory refinement based on spatio-temporal constraints

The spatio-temporal constrains should be applied during the route generation process to filter out the real possible trajectories. At first, we apply a time window as temporal constraints to determine the start and end of the trajectory. For the PIR sensor data, a time window of 3 s is suitable to segment the trajectories as individual trajectory. With this configuration, we can separate the responses of the time constraints during the route extension. The spatial constraints are determined by the indoor topological structure and the relations between the tracked nodes. Here, we query any active neighborhood node in the next time window and construct the spatial constraint matrix ***C***. The spatial constraint matrix ***C*** is a diagonal matrix, where *e_k_* are the possible active sensor nodes, and we define ***C_kk_*** = 1, otherwise, the ***C_kk_*** = 0. Therefore, we can define the whole spatial constraint matrix as follows:

(9) $$C_{ij}^{n}=\{\begin{array}{l} 1,\;i=j,\;e_{i}\;is\;possible\;node\\ 0 \end{array}$$

To extract the trajectory, we apply moving window query to the sensor activation log from the temporal dimension. When there is sensor activation in the next time window, a spatial constraint matrix ***C*** is constructed, except when the node queried is considered the ending node of a trajectory. If there are some nodes that are active but not responded to neighborhood nodes, we consider the nodes the new starting nodes of a trajectory. According to the new starting nodes, we can construct a new judgement matrix ***T***, which is also a diagonal matrix, where the element in the new judgement matrix ***T*** defines the new starting node *e_i_*. The construction rule of the new judgement matrix ***T*** is as follows:

(10) $$T_{ij}^{n}=\{\begin{array}{l} e_{i},\;i=j,\;e_{i}\;is\;the\;response\;node\\ 0 \end{array}$$

According to the Equations (9) and (10), *n* is the same with the network adjacent matrix. With the construction of the spatial constraints matrix ***C*** and judgement matrix ***T***, we can multiply the adjacent matrix ***M***, the spatial constraint matrix ***C*** and the judgement matrix ***T*** to construct the new adjacent matrix ***M’*** in this matrix. The non-zero element is the real motion trajectory in the *n*-th order. This can be formulated as:

(11) $$M^{\prime }=M\cup C^{n}+T^{n}$$

(2) The human motion trajectory sequence based route extension

The route extension is based on the route expanding using the oriented join product based on the GA-representation of the sensor networks. The adjacent relations between different nodes are inherited in ***M***. For the given sensor activation sequence $\left\{X\}=\{X_{1},\cdots ,X_{2},\cdots ,X_{n}\right\}$, the *n*-order adjacent matrix can be extended as:

(12) $$M^{\prime n}={M^{\prime }}_{F\{x_{1}\}T\{x_{2}\}}\cup {M^{\prime }}_{F\{x_{2}\}T\{x_{3}\}}\cup \cdots \cup {M^{\prime }}_{F\{x_{n-1}\}T\{x_{n}\}}$$

To covert the matrix into the *n*-order routes, the starting node matrix ***Q*** is defined here. ***Q*** is a diagonal matrix, in which the *i*-th row and column has the elements of $e_{i}$, which means all the routes have the starting node of $e_{i}$. The construction of the starting node matrix ***Q*** is as follows:

(13) $$Q_{ij}=\{\begin{array}{l} e_{i}\;i=j,\;e_{i}\;is\;the\;starting\;node\\ 0 \end{array}$$

According to the above route extension and construction rule, we can then realize the spatial trajectory reconstruction according to the sensor log data. The reconstruction is defined according to the following equations:

(14) $$\{\begin{array}{l} A^{2}=Q\cup M_{F\{x_{1}\}T\{x_{2}\}}\\ A^{n}=Q\cup A^{n-1}\cup M_{F\{x_{n-1}\}T\{x_{n}\}} \end{array}$$

### 3.3. Possible Trajectories Generation and Refinement Algorithm

**Figure 6:**
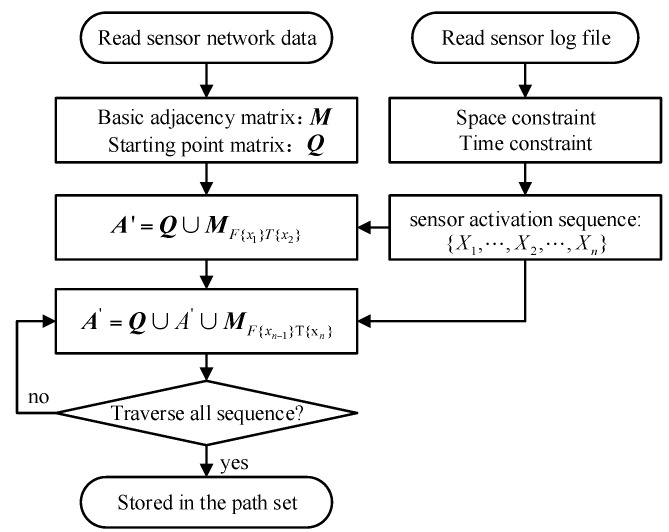
The process of the trajectory generation and refinement algorithm.

Based on the above definitions, we can then develop a unified algorithm to generate and refine all the possible trajectories according to the sensor activation log. The algorithm uses the oriented join product to realize the route extension and uses the spatial and temporal constraints to refine the generated trajectories. The algorithm starts with the fundamental adjacent matrix ***M*** that is constructed according to the topology of the sensor network. Clearly, the multiplication of the matrix ***M*** can produce all possible routes that the human can walk in the geographical space. According to the sensor network activation data, the starting nodes of the trajectories are first queried to construct the starting node matrix Q. All feasible trajectories are contained in the matrix ***M****^n^*. Then the spatial and temporal constraints can be applied to filter the high order matrix ***M****^n^* to extract the more accurate trajectories. By determining the completeness of a trajectory and repeating the trajectory generation process, all the possible trajectories can be extracted. The overall process of the algorithm is illustrated in the Figure 6.

The example of the simple sensor network depicted in Figure 1a is used to illustrate the algorithm. In the Figure 1a, every sensor represents the sensor activation of the human motion. Assuming the sensor response sequence is A(0s~1s), C(2s~4s), D(2s~3s), B(4s~5s), E(4s~6s), F(5s~6s), the reconstruction process of the network trajectory is: 

(1) According to the time constraint (3s) of human motion trajectory, the sensor activation sequences is {A}→{C,D}→{B,C,E}→{E,F}. Therefore, the human motion trajectory reconstructing equation is:

(15) $$\left\{ \begin{array}{l} A^{2}=Q\cup M_{F\{A\}T\{C,D\}}\\ A^{3}=A^{2}\cup M_{F\{C,D\}T\{B,C,E\}}\\ A^{4}=A^{3}\cup M_{F\{B,C,E\}T\{E,F\}} \end{array}\right.$$

(2) Introducing the starting node matrix ***Q***, and adjacent matrix ***M***, Equation (15) can be written as:

(16) $$\left\{ \begin{array}{l} A^{2}=Q\cup \left|\begin{array}{ll} e_{C} & 0 \end{array}\right|\\ A^{3}=A^{2}\cup \left|\begin{array}{lll} e_{B} & e_{C} & 0\\ 0 & e_{C} & e_{E} \end{array}\right|=Q\cup \left|\begin{array}{lll} e_{CB} & e_{CC} & 0 \end{array}\right|\\ A^{4}=A^{3}\cup \left|\begin{array}{ll} 0 & 0\\ 0 & e_{F}\\ e_{E} & 0 \end{array}\right|=Q\cup \left|\begin{array}{ll} 0 & e_{CCF} \end{array}\right| \end{array}\right.$$

Multiply the starting node matrix ***Q***, we can get the *n*-order routes:

(17) $$\left\{ \begin{array}{l} A^{2}=e_{AC}\\ A^{3}=e_{ACB}+e_{ACC}\\ A^{4}=e_{ACCF} \end{array}\right.$$

(3) The routes in Equation (17) can be classification according to the activated sensor sequences expresses in Equation (16). Firstly, since the response matrix of 2-order routes are diagonal matrix, the results are continuous *moving* trajectory; in the response matrix of 3-order routes, the matrix $\left|\begin{array}{ll} / & e_{C}\\ / & e_{C} \end{array}\right|$ meet the condition of *split* trajectories. So, in the 3-order routes there exist *split* trajectories *e_ACB_* and *e_ACC_*. At the same time, the *e_CC_* is the diagonal element of the adjacency matrix ***M***, *e_C_* is also a *still* node here; in the response matrix of 4-order routes, there are not any special structure, so it only inherited the *still* node *e_C_* of 3-order routes.

Clearly, the algorithm we proposed is automated and is clearer to represent the real and possible trajectories from the sensor log data. The spatial constraints, network topologies and the route uncertainties are largely reduced. The route extension is dynamic and generative, which can also be interactively updated. All the possible trajectories can be extracted algebraically, which can be simply computed and analyzed in the future.

## 4. Case Studies

**Figure 7:**
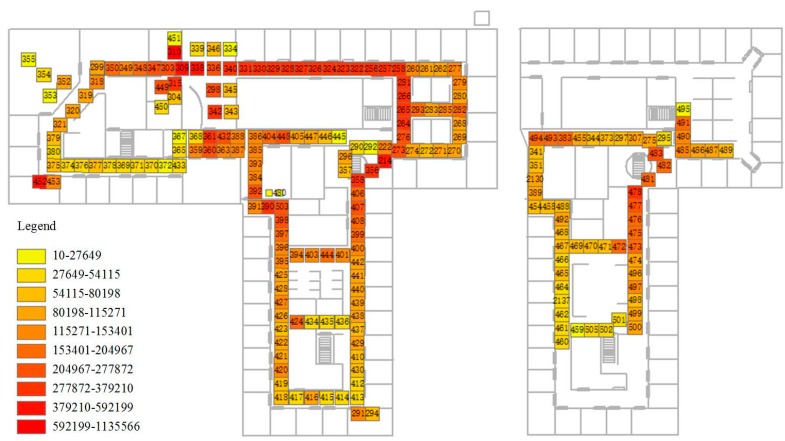
Spatial distribution of the sensor activation.

The algorithm is applied to the MERL sensor database published by the Mitsubishi Electric Research Labs. There are a total of 213 sensors, which logged the human motion in the building from 21st May 2006 to 24th March 2007 and were distributed on the 7th and 8th floor [8]. The spatial distribution of the sensors is depicted in the Figure 7. The sensors are installed with the intention of covering the floor area completely with little or no overlap between sensor fields of view. Although the minimum inter-detection time varies, the average minimum inter-detection time is around 1.5 s. The event log of the Lab is also provided as reference data for validation. The computation is performed on an Inspur NP 3560 server with two Intel Xeon E5645 (2.4 G) processor, 48 GB DDR-3 ECC Memory and a Raid five disk volume made up of three Seagate ST4000NM0023 SAS hard disk (7200 RPM). The operation system is Windows Server 2008 R2. All the data are imported in a PostgreSQL v9.4 database. The algorithm is implemented as a plug-in of the system CAUSTA [25] with ODBC connection to the PostgreSQL database server. To make better comparison with our method, the trajectory results extracted by the tracklet graph model is also used and imported in the database.

Firstly, the overall active frequency is summarized to get the spatial distribution of the active sensors (Figure 7). The hotspot graph suggests that the hottest area is around sensor 310, which is in the kitchen, because every workday people walk from various directions to the kitchen. Other hotspot regions are mostly meeting rooms (e.g., Belady Meeting Room (452)). The Belady meeting room is the most commonly used, based on the evidence from the activity log. Other frequently active sensors such as 255 and 356 are the ladders people frequently use. Therefore, from the sensor activation data, the spatial distribution of the human walking can be summarized. However, the statistics cannot reveal further trajectory information about people’s walking.

Since generating all possible trajectories from the whole data set is too complex, we selected one week of data of the 8th floor from 7 August to 13 August to generate all the possible people trajectories from the sensor activation data. There are totally 153 sensors and the total record of the active sensor log is 414,552. Based on the trajectory reconstruction algorithm, we have reconstructed 414,552 possible sensor statues and 563,386 total trajectories. After splitting the trajectories in a one-minute window and removing the duplicated trajectories, which have totally same nodes, we finally have 202,700 complete different trajectories.

The overall time cost of both the computation of the statues of each sensor and generation of all possible trajectories is 3 h and 19 min and the peak memory cost is 3.2 GB. The final result database file (including the original query data and generated path data) occupies a space of 3.07 GB in the NTFS file system. To test the correctness and performance of the method, we use the version 2 tracklets data (which is generated by the tracklet graph model at 22 March 2008, and published together as the reference data along with the MERL sensor data for the IEEE Information Visualization Challenge 2008) as the reference data. Since the original performance of the tracklet graph model are not possible to be accessed, we apply our method onto different numbers of sensor activation logs to evaluate the performance of the method.

The time and memory cost of the generation of the sensor status and trajectories from the start to different end time are logged during the computation (Table 1). The computation time is less than two and a half hours of the whole week’s data. 

**Table 1:** The computation performance evaluation (Start from 2006/August/7 0:00).

Time Range (End Time)	No. of Sensor Logs	Time Cost of Sensor Status(s) *	Memory Cost of Sensor Status(MB)	Time Cost of Trajectory Generation(s) *	Memory Cost of Trajectory Generation (MB)
2006/August/7 16:10	53517	1014.89	200.11	47.12	312.31
2006/August/8 07:19	77031	1314.89	213.82	67.96	450.15
2006/August/8 20:00	152031	2919.82	420.32	134.37	888.07
2006/August/9 23:46	229309	3214.71	480.68	202.67	1338.53
2006/August/12 10:30	315130	4231.27	510.24	277.59	1839.47
2006/August/12 23:05	414397	8919.82	718.48	365.85	2419.09
2006/August/13 23:59	414552	8992.71	729.14	365.26	2419.77

* The time cost logged here not include the time of data I/O.

**Figure 8:**
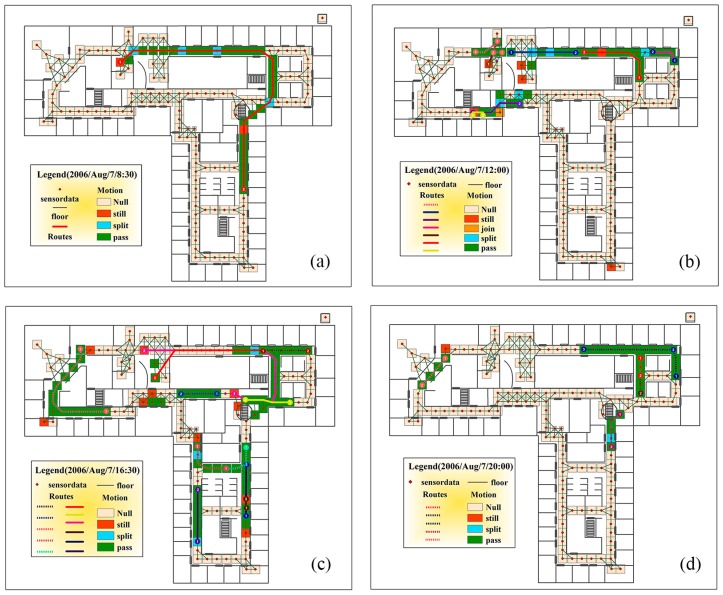
Route path and sensor status generated by our method compared with the path generated by the tracklet graph method in the same one minute. The solid line in the graph is the trajectory extracted by the tracklet graph model. The dashed line in the graph is the missing trajectories that have been extracted by our method but missing by the tracklet graph model. (**a**) Trajectory and sensor status extracted at 8:30; (**b**) Trajectory and sensor status extracted at 12:00; (**c**) Trajectory and sensor status extracted at 16:30; (**d**) Trajectory and sensor status extracted at 20:00.

Since in the sensor status determination, the oriented join products are not computed in realistic terms, but only the binary matrix is used to classify the *pass*, *join* and *split* status. The major performance bottleneck in this procedure may be the determination of the *still* and *stop* status. Not so restrictedly speaking, the time efficiency of the sensor status query is nearly increased as a linear relation of the total number of sensor logs. For the performance of the trajectory generation, the computation cost is growing much larger, this is because the generation of the trajectories requires one to compute the real oriented join product to generate the path. The generated path should also be compared with the spatial and temporal constraints of filtering the path. The optimization of the oriented join product and optimized data structure of the computation may largely improve the computation performance. Compared with the tracklet graph models proposed by [11], which extracted 105,757 tracklet graphs, our method can provide more complete possible human motions. This is because the tracklet graph model can only reveal one trajectory from a single sensor network active state [17,20]. The detailed comparison between our result and the tracklet graph method is illustrated in Figure 8. From Figure 8, we can clearly see both the extracted path and the sensor status in one minute of time, which is more semantically meaningful than the tracklet graph model. In addition, our method can provide more detailed trajectories that are not generated by the tracklet graph model, *i.e.*, our method can provide the join, split, still and pass status of each sensor to provide more detailed descriptions about the human motion. In the tracklet graph model result, several possible trajectories are not extracted (Table 2). There are also wrong trajectories extracted (e.g., trajectory from sensor id 257 to 342 in 16:30) by the tracklet graph model. Since the tracklet graph models are a statistical method that extract the start and end node of the trajectory using the graph cut statistics, the internal structures of the trajectory are not fully used to restrict the detailed analysis. Since the single sensor activation log data may lead to several different human motion trajectories, there must be several trajectories lost in the tracklet graph construction. From the result of our algorithm, we can extract all possible trajectories. Although not all the trajectories happened in the real world, there exists a possibility that these trajectories produce the active sensor pattern. Comparing and combining the use of the tracklet graphs and our data may produce interesting and more accurate human motion pattern results. It is also helpful to apply modern statistical methods to analyze the detailed pattern of the human motions [21,22].

**Table 2:** Absent trajectories that are missing compared with the tracklet graph method.

Time	Start Node (Sensor ID)	End Node (Sensor ID)
2006/August/7 12:00	309	348
2006/August/7 16:30	408	444
	444	398
	371	299
	405	386
	256	277
2006/August/7 20:00	265	276
	318	321
	356	408
	282	326
	281	265

**Figure 9:**
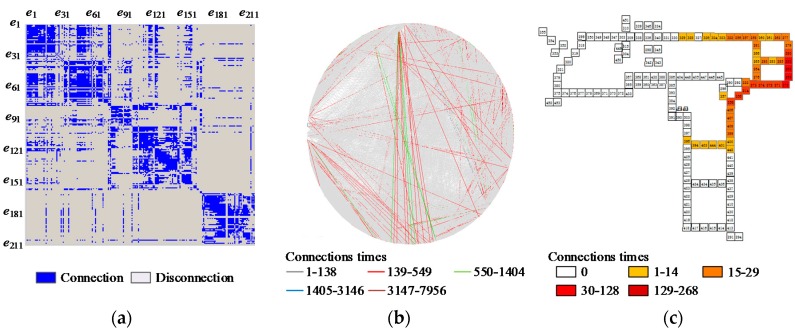
The start and end node analysis. (**a**) The matrix representation; (**b**) The circular representation; (**c**) End nodes for start sensor 272.

To further validate the correctness of our method, we counted all the trajectories by visualizing the start-end node connections using matrix and circular graph (Figure 9a,b). We conclude that most trajectories have similarities, *i.e.*, the people trajectory starting from the same sensor usually have some fixed ending nodes of the sensor. This is also the real case due to the work pattern in the office area. As everyone’s work has special tasks that may be connected with people who have working relations. To further reveal the trajectory patterns, we also provide the visualization of the spatial and frequency distribution of the ending nodes of the trajectories started with the same starting node. For example, starting from the sensor ID 272, we have totally 47 sensors that have been selected as ending nodes (Figure 9c). However, the most frequently linked sensors are neighborhood sensors, which suggests that people working in the office located at the sensor 272 mostly have working relations with the neighborhood office. This result can also be supported by similar analysis with the entropy method [15], which suggests the individuals associated with this sensor are frequently concentrated in the large office. From these statistical results, we believe that our method can be used to reveal the human motion patterns directly according to the sensor activation log data.

**Figure 10:**
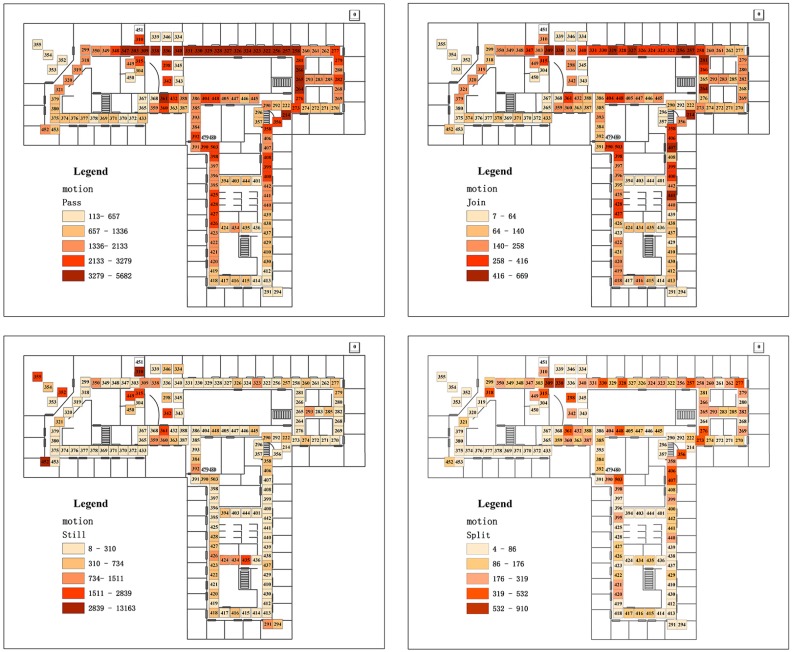
The statistics of the different behaviors.

With our method, the different states of the sensors according to the trajectories can also be extracted (Figure 10). Since the sensor nodes at different locations have different functions in the working place and the human passing patterns will be affected by the spatial topology of the network, the frequency and spatial distribution of the sensor states can also be evaluated by the sensor locations.

Four different states, including passing, join, split and still, are analyzed. The passing status has the most numbers, and the region of the highest passing frequency is in the line from sensor 348 to sensor 273. This area is the dining room and every noon people walk to this area to have lunch. For the join status, the highest frequency happens at sensors 309, 338, 331, 330, 329, 327, 326, 323, 322, 256, and 257. These places are near the dining room. People from different offices join their trajectories to have lunch. Other individual high frequency join statuses happen at sensors 214, 407, 441, 281, and 264; these sensors are located near the cross where people from different directions join their trajectories. In [15], sensor 442 is classified as the hub that connects different groups of people. In our result, the statements of [15] can also be partially supported. However, we can filter out more such kinds of hubs from the possible trajectories. In addition, the location of the hub is slightly different from the entropy method. Since our result is directly generated according to the spatial topology structure and the sensor activation log, which also integrates different spatio-temporal constraints, it may be more accurate than the statistical segmentation which uses the entropy. For the sensors with status of split, highest frequency sensors are located at the corner or across the work place. The exceptions are sensor 295, 330, 328, 324 and 323. These five sensors are near the dining room, where people walk to their own offices after lunch. The sensors with high frequency of still status include 355, 352, 452, 449, 315, 342, 261, 301 and 435. Among them the sensors 355 and 352 are the Nitta seminar room; sensor 452 is the Belady conference room; sensors 449 and 315 are mail and supply rooms. Sensors 342 and 261 are restrooms; sensor 301 is a lunch room; sensor 435 are stairs to the 7th floor. According to the function of these locations of the sensors, especially for the mail and supply rooms and restrooms, people are definitely more likely to stand still here instead of stopping their trajectories. The above results further validate the correctness of our method.

## 5. Discussion and Conclusions

In our research, the spatial and temporal constraints play a key role. The major uncertainty of our method is the time interval used to determine the starting and the ending nodes of the trajectories. Future analysis can be applied in the direction of what’s the sensitivity of the time windows. Although we have generated all the possible trajectories from the sensor activation data, not all the possible differences between different trajectories are considered. The long-term data observation and the detailed lab event log data make the statistical inference of the possibility difference of each reconstructed trajectory possible. For example, the reconstructed trajectories can be further reanalyzed by the HMM, LDA, Bayesian filtering or temporal segment methods [2,21,22]. The analysis based on the refined trajectories data may largely improve the accuracy and conclusion ability of such researches. Further applications in smart homes or buildings can also benefit from our method.

The GA-based route generation and constraint integration method provide unified and flexible tools for network and trajectory analysis. In the GA-based approach, the route generation is based on the matrix approach according to the topological information of the original network. The approach makes the stepwise route generation of each possible route very flexible. Although the matrix-based storage is memory and computational inefficient, a lot of optimizations can be applied to support the large-scale analysis. Since the route generation is symbolic and independent of other orders of routes, the pre-complier and parallel computation (e.g., Gaalop) technologies may be applied to help improve the efficiency [33,34,35]. In addition, we can also develop specialized data structures for online data stream computation for the passive sensor networks such as the MERL sensor data sets. Tensors and other compression method may also be helpful for the data representation and analysis [36,37].

In our GA-based trajectory reconstruction method, the route extension is not only the real geographical spatio-temporal sequence, but also an algebraical element that can be directly calculated. The clear geometric and physical meaning of the motion can be directly revealed from the algebraical equations. In this way, the representation and analysis method can make the analysis of the motions much simpler and clearer. Since both the outer product and the oriented join product is asymmetric, the route extension has its own orientations, which can be well classified by the spatial and temporal adjacent relations in the sensor activation log. Since the orientation information is very important for the activity identification, our GA method may also be helpful to improve the activity recognition from the sensor data. According to the orientation information, we can reveal not only the classical activity of walking, entering, joining and splitting, but also the activities like turning left and turning right *etc.* This further detailed analysis may make the GA-based activity analysis a wider potential area. Since the GA representation and analysis is inherently high dimensional, the representation and analysis can be made simple and direct.

In this paper, we developed a human trajectory refinement method to reveal all the possible human motions from the passive MERL sensor activation log data. Our method unifies the representation of the sensor network, sensor activation data and the human moving trajectory under a unified mathematical framework. All the possible human motion trajectories are tracked according to the dense sensor activation log using the matrix approach. The geometric algebra can well express the absolute and relative coordinates of the human motion. The network and trajectory representation can well express the network and trajectory information in a unified multivector structure. The spatio-temporal constraints as well as the sequence information can be unitedly represented using the outer product. With our method, not only can all the possible trajectories of human motion be extracted, but also the spatial and temporal constraints can be flexibly applied during the route extension. The extracted motion trajectories can more accurately reflect the real human motion in the office environment. Our method provides a new solution that can deal with the uncertain problem of the trajectory reconstruction from the sensor network data. Further integration of our method and the statistical inference method may provide new possibility in passive sensor analysis. In addition, our method is also useful for sensor-network-guided indoor navigation and optimal routing.
